# Implementation of the AAMATES Platform as a National Registry for Stage 5 CKD in Mexico

**DOI:** 10.1016/j.xkme.2026.101396

**Published:** 2026-05-08

**Authors:** Rafael Valdez-Ortiz, Rodolfo Rincón Pedrero, Enzo Vásquez Jiménez, Joana Balderas Juárez, Ana Cecilia Navarro Ramírez, José Carlos Romo Vázquez, Bertha Córdova Sánchez, Gustavo Alejandro Casas Aparicio, Anarinka Saldaña Mendoza, Paulina Montserrat Muñoz Pérez, Gustavo Reyes Terán, Magdalena Madero

**Affiliations:** 1 Nephrology Department, Hospital General de México “Dr. Eduardo Liceaga”, Mexico City, Mexico; 2 Nephrology and Mineral Metabolism Department, Instituto Nacional de Ciencias Médicas y Nutrición Salvador Zubirán, Mexico City, Mexico; 3 Nephrology Service, Hospital Juárez de México, Mexico City, Mexico; 4 Nephrology Service, Hospital Gea González, Mexico City, Mexico; 5 Pediatric Nephrology Department, Instituto Nacional de Pediatría, Mexico City, Mexico; 6 Nephrology Department, Hospital Infantil de México Federico Gómez, Mexico City, Mexico; 7 Intensive Care Unit, Instituto Nacional de Cancerología, Mexico City, Mexico; 8 Nephrology Department, Instituto Nacional de Enfermedades Respiratorias, Mexico City, Mexico; 9 Medical Directorate, Instituto de Seguridad y Servicios Sociales de los Trabajadores del Estado (ISSSTE), Mexico City, Mexico; 10 Nephrology Department, Instituto Nacional de Cardiología Ignacio Chávez, Mexico City, Mexico

**Keywords:** chronic kidney disease, Mexico, national registry

## Abstract

**Rationale & Objective:**

Chronic kidney disease (CKD) poses a major public health challenge in Mexico. Despite this burden, no national registry exists, hindering an accurate understanding of the disease. This study sought to characterize patients with stage 5 CKD using the Administration and Management Environment for Health Services (AAMATES) digital platform and assess its potential as a national registry model.

**Study Design:**

A cross-sectional observational registry collected demographic and clinical data for stage 5 CKD through the AAMATES platform.

**Setting & Population:**

Patients treated between January and December 2023 at hospitals and institutes affiliated with Mexico’s Coordinating Commission of National Institutes of Health and High-Specialty Hospitals (CCINSHAE) were included. The registry captured 969 patients; mean age was 49.8 years and 56% were male.

**Exposures or Predictors:**

The main etiologies of CKD were type 2 diabetes mellitus (37.1%) and unknown causes (23.1%). Initial renal replacement therapy (RRT) was also documented: hemodialysis in 55%, peritoneal dialysis in 38%, preemptive transplant in 2%, and no RRT in 5%. Additional variables included residual urine volume and laboratory values (creatinine, hemoglobin, sodium, potassium, calcium, phosphorus, and albumin).

**Outcomes:**

Outcomes comprised the distribution and intensity of RRT, residual urine output, biochemical parameters, time from CKD diagnosis to registry entry, and whether patients had been evaluated by a nephrologist.

**Analytical Approach:**

Descriptive statistics were used to summarize the collected data, presented as frequencies, means ± standard deviations, and medians with interquartile ranges.

**Results:**

Among 537 patients receiving hemodialysis, 71% received ≤2 sessions per week; and among 365 patients receiving peritoneal dialysis, 32% received intermittent peritoneal dialysis. Residual urine volumes of 100-500 mL/d were observed in 46.6% of participants; the median time from CKD diagnosis to registry entry was 282 days, and 14% had not been evaluated by a nephrologist at diagnosis.

**Limitations:**

The registry is voluntary, is limited to stage 5 CKD, lacks data on time since diagnosis for nearly half of participants, and includes only CCINSHAE hospitals, excluding other major Mexican health care systems.

**Conclusions:**

AAMATES demonstrates CCINSHAE’s effort to map advanced CKD. Making this registry mandatory could enable robust epidemiological monitoring and targeted public health strategies to lessen Mexico’s CKD burden.

## Plain-Language Summary

Chronic kidney disease stage 5 represents a major public health challenge in Mexico, yet national data remain limited. This study describes the implementation of AAMATES, a digital platform designed to function as a national registry for patients with advanced kidney disease. The registry collects clinical, demographic, and treatment-related information from participating institutions across Mexico. Our findings demonstrate that a national kidney disease registry is feasible and may help improve epidemiological surveillance, healthcare planning, resource allocation, and future public health strategies for kidney disease in Mexico.

## Introduction

Chronic kidney disease (CKD) is a major public health concern in Mexico.^1^ Contributing to the high burden of noncommunicable diseases, CKD incidence is expected to rise unless effective prevention strategies are implemented.^2^ National disease registries are essential tools for understanding epidemiology, guiding health investments, mapping patient care trajectories, and identifying barriers to access.^3^ CKD in Mexico poses significant challenges due to the high cost of renal replacement therapy (RRT) and the lack of a universal health care system, which leads to inequities in treatment access and insurance coverage.^4^, ^5^, ^6^

Since the late 20th century, the importance of national health registries has been widely recognized.^7^ To be effective, such registries must meet technical, legal, and ethical standards, particularly in data protection and health system improvement.^8^ Government coordination is critical to ensure data security, confidentiality, and resilience against unauthorized access or data loss.^9^

In Mexico, the Ministry of Health, through the General Directorate of Information Technologies and the Health Information Directorate, developed and implemented a digital platform named Administration and Management Environment for Health Services (AAMATES). Its purpose is to digitize clinical data across hospitals, facilitate record-keeping and interinstitutional communication, and reduce information gaps across all levels of care.^10^

In 2023, the Coordinating Commission of National Institutes of Health and High-Specialty Hospitals (CCINSHAE) initiated the development of a registry of patients with stage 5 CKD within the AAMATES platform. This represents an institutional and governmental effort to generate national data on CKD and inform public health diagnostics and treatment strategies. The objective of this publication is to describe the characteristics of patients with stage 5 CKD using the AAMATES platform as a model for a national CKD registry. Data were collected from 13 hospitals and institutes affiliated with CCINSHAE. In the absence of a universal health system in Mexico,^11^ AAMATES offers a digital strategy to improve coordination and interoperability among diverse health care subsystems.^12^

## Methods

### Study Setting and Design

**Figure 1:**
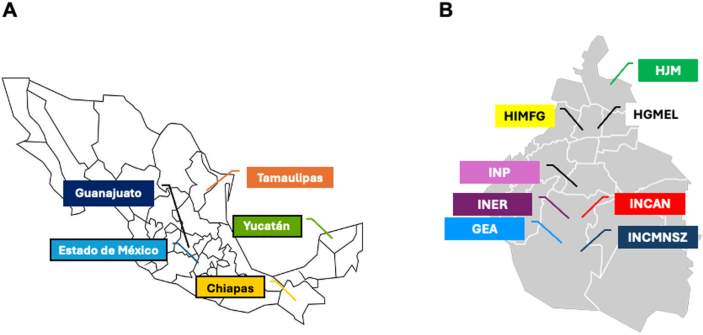
Geolocation map of participating hospitals and institutes. (A) Participating hospitals across Mexico included: Hospital Regional de Alta Especialidad de Ciudad Victoria, Tamaulipas (orange); Hospital Regional de Alta Especialidad del Bajío in Guanajuato (dark blue); Hospital Regional de Alta Especialidad Ciudad Salud in Chiapas (yellow); Hospital Regional de Alta Especialidad of the Yucatán Peninsula (green); and Hospital Regional de Alta Especialidad de Ixtapaluca (light blue). (B) In Mexico City, participating institutions included: Instituto Nacional de Ciencias Médicas y Nutrición Salvador Zubirán (INCMNSZ); Instituto Nacional de Cancerología (INCAN); Instituto Nacional de Enfermedades Respiratorias (INER); Hospital General Gea González (GEA); Instituto Nacional de Pediatría (INP); Hospital General de México “Dr. Eduardo Liceaga” (HGMEL); Hospital Infantil de México Federico Gómez (HIMFG); and Hospital Juárez de México (HJM).

This cross-sectional study included patients with stage 5 CKD (KDIGO [Kidney Disease: Improving Global Outcomes] criteria)^13^ who received care between January and December 2023 at 13 CCINSHAE-affiliated national hospitals and institutes (8 in Mexico City and 1 each in the States of Mexico, Yucatán, Chiapas, Tamaulipas, and Guanajuato) (Fig 1). Patients had an estimated glomerular filtration rate <15 mL/min/1.73 m^2^, with or without RRT.

### Data Collection Procedures

A structured registry was built into the AAMATES platform (website https://aamates.salud.gob.mx/admin/) including: (1) primary CKD cause (diabetes, hypertension, congenital anomalies of the kidney and urinary tract, primary or secondary glomerulopathies, unknown, other) defined by the treating nephrologist at enrollment based on clinical records, laboratory data, imaging, and histopathology when available; cases without a definitive diagnosis were classified as unknown etiology; (2) prior RRT (yes/no); (3) RRT initiation date (month/year); (4) current RRT modality (hemodialysis [HD] 1, 2, or 3 times weekly; continuous ambulatory peritoneal dialysis; automated peritoneal dialysis; or intermittent peritoneal dialysis [IPD]); patients not receiving RRT were classified separately as CKD stage 5 without RRT; (5) residual urine volume (RUV): >500 mL/d, 100-500 mL/d, <100 mL/d; (6) comorbid conditions (hypertension, diabetes, cardiovascular disease, smoking, cancer, stroke, autoimmune disorders); (7) laboratory measurements at admission (hemoglobin, creatinine, sodium, potassium, calcium, phosphorus, albumin); (8) body mass index; and (9) prior nephrology consultation. Data entry into the AAMATES platform was performed by administrative staff and medical trainees at each center. Because some noncritical fields are not mandatory, completeness varied among variables, reflecting operational variability rather than systematic clinical patterns.

### Data Analysis

Descriptive statistics were used. Normally distributed variables were expressed as mean ± standard deviation and nonnormally distributed variables as median and interquartile range. SPSS version 25 (IBM Corp) was used for analysis.

### Ethics Statement

Patients were enrolled according to the principles of the Declaration of Helsinki (latest revision, Fortaleza 2013). Upon admission to CCINSHAE institutions, patients provided informed consent for the use of clinical data in research, ensuring confidentiality and data privacy.

## Results

### Clinical and Demographic Characteristics

A total of 969 patients were registered, with a mean age of 49.8 ± 16.2 years; 544 (56%) were male. Baseline characteristics are shown in Table 1. Marked heterogeneity was observed across treatment groups. Patients with CKD managed without RRT, those receiving HD thrice weekly, and those receiving continuous ambulatory peritoneal dialysis were older, whereas those receiving automated peritoneal dialysis and IPD included younger individuals (*P* < 0.01). Major comorbid conditions, including hypertension and diabetes, differed significantly across modalities (*P* < 0.001). RUV showed a pronounced gradient (*P* < 0.001), with complete preservation in CKD stage 5 without RRT and severe oliguria predominating in those receiving HD thrice weekly. Key laboratory parameters also differed significantly between groups (*P* < 0.001), and lack of nephrology assessment at CKD stage 5 diagnosis was most frequent in patients receiving IPD (*P* < 0.001), underscoring disparities in access to specialized care. Twenty-seven patients were younger than 18 years, with a median age of 13 years (range, 8-17 years).

**Table 1:** Baseline Clinical, Demographic, and Laboratory Characteristics According to Kidney Replacement Therapy Modality

Variable	Total	CKD G5 Without RRT	HD Once Weekly	HD Twice Weekly	HD Thrice Weekly	CAPD	APD	IPD	*P* value
	N = 969 (100%)	67 (7%)	57 (6%)	325 (33.5%)	155 (16%)	209 (21.5%)	38 (4%)	118 (12%)	
Age (y), mean ± SD	49.8 ± 16.1	54.2 ± 17.5	44 ± 12.4	46.1 ± 11.2	51.61 ± 14.8	56.1 ± 13.5	42.7 ± 14.3	41.9 ± 10.8	<0.01
Men, n (%)	544 (56%)	40 (59%)	32 (56%)	165 (51%)	85 (55%)	128 (61%)	21 (55%)	73 (62%)	0.020
Comorbid conditions, n (%)									
High blood pressure	722 (75%)	67 (100%)	50 (88%)	223 (68%)	127 (82%)	188 (90%)	23 (61%)	44 (37%)	<0.001
Diabetes mellitus	408 (42%)	45 (67%)	43 (75%)	110 (34%)	89 (57%)	79 (38%)	12 (32%)	30 (25%)	<0.001
Cardiovascular diseases	71 (7%)	2 (3%)	4 (7%)	7 (2%)	12 (8%)	26 (7%)	5 (13%)	15 (13%)	<0.001
Smoking	70 (7%)	4 (6%)	8 (14%)	12 (4%)	16 (10%)	13 (6%)	4 (11%)	13 (11%)	0.014
Cancer	19 (2%)	8 (12%)	1 (2%)	3 (1%)	4 (3%)	2 (1%)	0 (0%)	1 (1%)	<0.001
Cerebral vascular disease	13 (1%)	0 (0%)	1 (2%)	2 (1%)	3 (2%)	3 (1%)	0 (0%)	4 (3%)	0.324
Autoimmune diseases	61 (6%)	0 (0%)	2 (4%)	23 (7%)	17 (11%)	6 (3%)	1 (3%)	12 (10%)	0.004
Body mass index^a^ (kg/m^2^) mean ± SD	23.9 ± 4.88	25.1 ± 4.74	24.89 ± 5.95	23.01 ± 3.03	24 ± 4.25	24.99 ± 5.13	20.96 ± 5.02	22.09 ± 5.8	<0.001
Urinary volume^b^, n (%)									
<100 mL/24 h	218 (25%)	0 (0%)	9 (16.1%)	79 (27.1%)	68 (45%)	39 (20.5%)	79 (27.1%)	19 (20.4%)	<0.001
100-500 mL/24 h	407 (46.6%)	0 (0%)	26 (46.4%)	142 (48.8%)	67 (44.4%)	113 (59.5%)	142 (48.8%)	49 (52.7%)	<0.001
>500 mL/24 h	247 (28.4%)	66 (100%)	21 (37.5%)	70 (24.1%)	16 (10.6%)	38 (20%)	70 (24.1%)	25 (26.9%)	<0.001
Laboratory measurements, mean ± SD									
Serum hemoglobin (g/dL)	9.44 ± 2.24	10.82 ± 1.88	8.18 ± 1.70	9.44 ± 1.47	10.32 ± 2.25	9.83 ± 2.44	9.37 ± 3.72	7.45 ± 2.36	<0.001
Serum creatinine (mg/dL)	9.38 ± 5.54	6.26 ± 6.69	3.93 ± 3.92	5.22 ± 1.27	10.2 ± 1.71	12.5 ± 2.83	14.4 ± 4.6	16.9 ± 6.1	<0.001
Serum sodium (mmol/L)	136.3 ± 4.8	136.7 ± 5.1	136.4 ± 4.1	136.7 ± 3.9	136.7 ± 3.1	136.1 ± 4.1	136.2 ± 2.4	135.2 ± 4.8	0.350
Serum potassium (mmol/L)	4.7 ± 0.9	5.1 ± 0.71	4.7 ± 0.70	4.67 ± 0.69	4.7 ± 0.68	4.62 ± 0.66	4.48 ± 0.65	4.7 ± 0.71	<0.001
Serum calcium (mg/dL)	8.02 ± 1.17	9.17 ± 0.76	7.89 ± 1.1	8.06 ± 0.98	7.95 ± 1.12	7.87 ± 1.32	8.16 ± 1.42	7.79 ± 1.12	<0.001
Serum phosphorus (mg/dL)	5.36 ± 2.22	6.34 ± 2.86	6.23 ± 1.99	5.6 ± 2.04	4.88 ± 1.82	5.04 ± 1.82	3.74 ± 1.43	5.49 ± 2.94	<0.001
Serum albumin (g/dL)	3.41 ± 0.78	3.65 ± 0.80	2.86 ± 0.93	3.63 ± 0.47	3.44 ± 0.70	3.56 ± 0.76	3.52 ± 0.57	3.48 ± 0.84	<0.001
Time to diagnosis of CKD G5^c^ (d), median (IQR)	282 (69.25-673.5)	221 (98.9-537)	254 (106.1-478.8)	316 (114-712)	306 (91-629)	240 (85-593)	340 (181.3-705.2)	311 (105.4-711.3)	0.535
No nephrologist assessments at CKD G5 diagnosis, n (%)	138 (14.24%)	0 (0%)	18 (27%)	21 (6%)	9 (6%)	32 (15%)	1 (3%)	57 (48%)	<0.001

Abbreviations: APD, ambulatory peritoneal dialysis; CAPD, continuous ambulatory peritoneal dialysis; CKD G5, chronic kidney disease stage 5; HD, hemodialysis; IPD, intermittent peritoneal dialysis; IQR, interquartile range; RRT, renal replacement therapy; SD, standard deviation.

a Averages were calculated from complete data for 770 patients.

b Information obtained from 872 patients.

c Median and IQR values were based on 372 patients.

**Figure 2:**
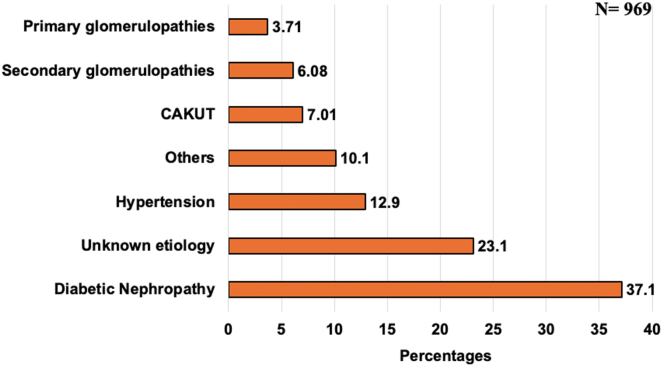
Causes of stage 5 chronic kidney disease (KDIGO [Kidney Disease: Improving Global Outcomes]). Percent distribution: diabetic nephropathy (37.1%), unknown etiology (23.1%), hypertension (12.9%), other causes (10.1%), congenital anomalies of the kidney and urinary tract [CAKUT] (7.01%), secondary glomerulopathies (6.08%), and primary glomerulopathies (3.71%).

The primary causes of CKD were type 2 diabetes (359 patients, 37.1%), unknown etiology (224, 23.1%), hypertension (125, 12.9%), other causes (98, 10.1%), congenital anomalies of the kidney and urinary tract (68, 7.01%), secondary glomerulopathies (59, 6.08%), and primary glomerulopathies (36, 3.71%) (Fig 2). Among the ‘other causes’ group, the following were identified: 33 cases of obstructive uropathy with chronic vesicoureteral reflux (3%), 17 failed kidney grafts (2%), 16 cases each of tubulointerstitial nephritis or exposure to nephrotoxins (2%) and autosomal dominant polycystic kidney disease (2%), 5 cases of gout and hyperuricemia (1%), 2 patients with a solitary kidney (0.2%), 2 patients with type 1 cardiorenal syndrome (0.2%), and 1 case each of Joubert syndrome, Kasabach-Merritt syndrome, oculorenal syndrome, glycogen storage disease type I, primary hyperoxaluria, and non-Hodgkin lymphoma infiltration (all <0.2%).

Among the 537 patients receiving HD, 325 (61%) received 2 sessions per week, 155 (29%) received 3 sessions per week, and 57 (10%) received 1 session per week. HD frequency did not differ significantly according to geographic origin (Mexico City vs other regions), with similar proportions of patients receiving twice and thrice weekly HD. Regarding peritoneal dialysis, of the 365 patients, 209 patients (57%) were treated with continuous ambulatory peritoneal dialysis, 118 (32%) with IPD, and 38 (11%) with automated peritoneal dialysis.

### RUV and RRT

**Figure 3:**
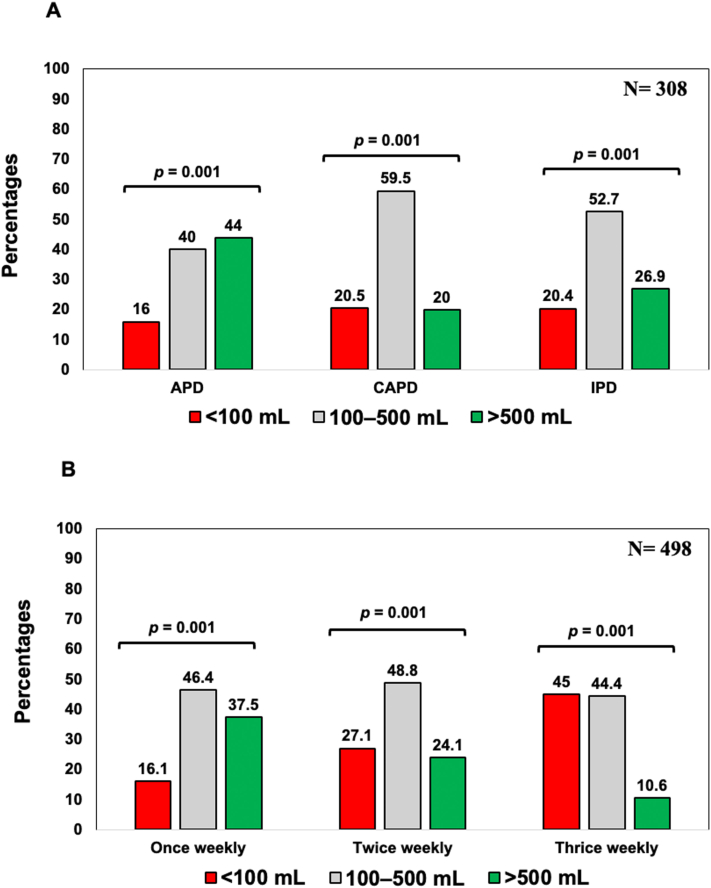
Residual urine volume (RUV) and type of renal replacement therapy. Colors represent: <100 mL/d (red); 100-500 mL/d (gray); >500 mL/day (green). (A) Peritoneal dialysis: among patients receiving APD, 61% had RUV >500 mL/d (*P* = 0.001); among those receiving CAPD, 57% had RUV 100-500 mL/d (*P* = 0.001); among those receiving IPD, 18% had RUV <100 mL/d (*P* = 0.001). (B) Hemodialysis: in patients undergoing ≤1 session/wk, 46% had RUV 100-500 mL/d (*P* = 0.001); among those receiving 2 sessions/wk, 45% had RUV 100-500 mL/d (*P* = 0.001); and among those receiving 3 sessions/wk, 11% had RUV >500 mL/d (*P* = 0.001). APD, ambulatory peritoneal dialysis; CAPD, continuous ambulatory peritoneal dialysis; IPD, intermittent peritoneal dialysis.

Among the 872 patients with recorded RUV, 308 were receiving peritoneal dialysis, 498 HD, and 66 with CKD stage 5 without RRT. Figure 3A shows the distribution of RUV among peritoneal dialysis modalities. Among patients receiving automated peritoneal dialysis, 4 (16%) had <100 mL/d, 10 (40%) had 100-500 mL/d, and 11 (44%) had >500 mL/d (*P* = 0.001). Among those receiving continuous ambulatory peritoneal dialysis, 39 (20.5%) had <100 mL/d, 113 (59.5%) had 100-500 mL/d, and 38 (20%) had >500 mL/d (*P* = 0.001). Of the patients receiving IPD, 19 (20.4%) had <100 mL/d, 49 (52.7%) had 100-500 mL/d, and 25 (26.9%) had >500 mL/d (*P* = 0.001). Figure 3B illustrates the RUV distribution among HD frequencies. Of those receiving HD 3 times per week, 68 (45%) had <100 mL/d, 68 (44.4%) had 100-500 mL/d, and 16 (10.6%) had >500 mL/d (*P* = 0.001). Of those undergoing twice weekly HD, 79 (27.1%) had <100 mL/d, 142 (48.8%) had 100-500 mL/d, and 70 (24.1%) had >500 mL/d (*P* = 0.001). Of the patients receiving once weekly HD, 9 (16.1%) had <100 mL/d, 26 (46.4%) had 100-500 mL/d, and 22 (37.5%) had >500 mL/d (*P* = 0.001).

## Discussion

National health registries are essential tools for public health and medical research.^8^^,^^14^ Their implementation enables monitoring of disease incidence, prevalence, and trends, as well as assessment of public health interventions.^14^ In this context, the AAMATES platform has proven to be a valuable operational tool, allowing digital data recording across different hospitals and institutes. Our findings highlight significant deficiencies in the care of patients with CKD in Mexico. Valdez et al^5^ (2018) previously reported inequalities in access to RRT, but their study was based on data from a single Ministry of Health hospital. In contrast, the current registry includes 13 hospitals, all part of the CCINSHAE network, introducing a selection bias, as institutions such as Mexican Social Security Institute (Spanish acronym, IMSS) or Institute of Social Security and Services for State Workers (Spanish acronym, ISSSTE) are excluded, organizations in which care access and quality may differ.^15^

Clinically inadequate RRT schemes were identified. Among patients receiving HD, a considerable proportion received only 1 or 2 sessions per week, often without significant residual urine output, precluding classification as truly incremental therapy.^16^ In peritoneal dialysis, intermittent hospital-based regimens prevail, driven by symptom exacerbation, biochemical alterations, or institutional availability, compromising patient survival and straining inpatient resources.^5^ As previously described in the literature, these reflect persistent structural barriers to continuous, patient-centered care. In Mexico, access to RRT is largely determined by public social security systems, federal referral hospitals, state health services, and private out-of-pocket payment,^17^, ^18^, ^19^, ^20^, ^21^ compounded by Mexico’s limited health care funding, which remains below the regional average.^22^ However, because the AAMATES registry does not currently capture individual-level data on funding source, a direct association between suboptimal RRT and payment modality cannot be formally assessed. Consistent with prior evidence, a relevant finding is the high proportion of patients receiving ≤1 weekly HD session or IPD, practices that deviate from guideline-recommended therapy. These patterns reflect structural barriers within the Mexican health system—such as limited financing, insufficient infrastructure, and oversaturated dialysis units—rather than elective clinical decisions. Previous publications have documented how such systemic limitations contribute to delayed dialysis initiation, fragmented care, and adverse outcomes.^4^, ^5^, ^6^ Our observations are consistent with this evidence, showing that inadequate dialysis dose is associated with higher mortality, poorer metabolic control, and increased hospitalizations.

Importantly, the systematic collection of RUV represents a distinctive strength of the AAMATES registry. Unlike most end-stage renal disease registries worldwide, which do not routinely document RUV, its incorporation allows a more comprehensive evaluation of dialysis adequacy, volume control, and patient trajectories beyond conventional dialysis dose metrics.^23^, ^24^, ^25^, ^26^

Another important finding was the relatively low proportion of patients lacking prior nephrology evaluation—ie, those with late referral. This metric is associated with increased morbidity, mortality, and health care costs and is reported globally in 20%-50% of cases.^27^^,^^28^ However, the low frequency observed in our study should be interpreted cautiously, as key variables were not collected, such as number of primary care visits, timing of referral, or duration of nephrologist follow-up. With only 9.7 nephrologists per million population, this gap is particularly relevant in Mexico.^29^

We also documented a relatively young adult population with KDIGO stage 5 CKD, in contrast with that in high-income countries, where it predominantly affects individuals older than 60 years.^30^ This trend may be attributed to the early onset of noncommunicable diseases in Mexico, including diabetes, hypertension, and obesity, which accelerate CKD progression.^31^ Additionally, a high proportion of CKD cases lacked classical etiologies such as diabetes or hypertension, suggesting a high frequency of unknown causes. The 2021 Global Burden of Disease data indicated that many diagnoses are coded as ‘other’ or ‘unspecified’ due to limited etiologic work-up.^32^ In regions such as Aguascalientes, up to 60% of end-stage CKD cases were attributed to unknown causes—surpassing those linked to diabetes or urinary tract disorders. These findings underscore the urgent need for research on nontraditional CKD causes in Mexico, including genetic, environmental, and occupational factors.^31^ As an important clarification, although 23% of cases were classified as having an unknown etiology, the current pilot phase of the registry was not designed to collect environmental, occupational, genetic, or detailed clinical exposure variables that would enable meaningful subgroup analyses. This limitation highlights the need for future iterations of AAMATES to incorporate expanded etiologic modules, which would allow targeted investigation of emerging and nontraditional causes of CKD in Mexico.

Globally, Mexico lacks updated data on HD and peritoneal dialysis units.^32^ The International Society of Nephrology Global Kidney Health Atlas highlighted inequalities and heterogeneity in CKD care worldwide and emphasized the need to strengthen health information systems.^32^ In response, in 2023, the CCINSHAE convened a nephrologist working group to develop a public policy for integrated CKD care. This initiative was implemented in 2024, including acquisition of supplies and materials, improving access to RRT for both CKD and acute kidney injury.^33^ This model successfully standardized care across hospitals in Mexico City and its metropolitan area.^33^

In 2025, progress continued with the federal publication of the National Medical Care Protocols, prioritizing 6 lines of action: (1) overweight and obesity, (2) type 2 diabetes and metabolic syndrome, (3) hypertension, (4) CKD, (5) the first 1,000 days of life, and (6) lifelong vaccination.^34^ The National Medical Care Protocols aims to standardize preventive and therapeutic approaches across all levels of care, emphasizing early detection and pharmacologic management of CKD from the earliest stages.^34^

Although Mexico still lacks a formal national registry policy, the AAMATES platform provides an opportunity to develop disease-specific registries for chronic diseases, particularly CKD. Such registries would provide vital data to understand risk factors, evaluate treatment outcomes, and track disease progression. They would also support evidence-based public health planning, resource allocation, and policy development.

Among the limitations of our study, a major constraint is that the registry was restricted to CCINSHAE-affiliated hospitals and institutes, which introduces potential selection bias and limits the generalizability of our findings to other health care subsystems in Mexico—such as IMSS and ISSSTE—in which access, financing, organization, and quality of care differ substantially. This initial phase of AAMATES was intentionally conceived as a pilot to assess technical feasibility, variable standardization, and system operability within a relatively homogeneous institutional network. Nevertheless, this limitation highlights the need to expand and externally validate the registry across additional health care systems to advance toward a truly national platform that more accurately reflects the epidemiologic burden and care trajectories of stage 5 CKD in Mexico. Another important limitation is the proportion of missing data for certain variables, particularly the time from CKD diagnosis to registry entry (49%) and RUV or laboratory parameters (10%). These gaps primarily reflect operational variability in data entry, as these fields were not mandatory within the AAMATES platform and were completed by personnel with heterogeneous levels of training. This pattern suggests a mechanism consistent with missing at random as described by Little and Rubin,^35^ reflecting operational variability in data collection rather than systematic clinical differences. Although the study’s purely descriptive design limits the impact of this missingness on the overall interpretation of results, future iterations of the registry will require mandatory fields and strengthened quality-control processes to minimize incomplete data and ensure higher data reliability.

A key challenge identified is the need for trained personnel dedicated to data entry at each center. Greiver et al^36^ emphasized the impact of trained staff on improving data quality within disease registries in Toronto, Canada.^37^ In Mexico, where clinicians face heavy workloads and high burnout rates,^38^ assigning them administrative tasks such as data entry could compromise data quality and reliability. In response to these challenges, future phases of AAMATES will incorporate a formal quality-assurance framework. This will include the development of standardized training modules for personnel responsible for data entry, the incorporation of mandatory fields and automated validation checks to reduce inconsistencies at the point of capture, and periodic quality audits led by CCINSHAE technical committees. Continuous feedback mechanisms with participating centers through routine data quality reports, together with the progressive integration of AAMATES into institutional electronic health record systems, are also planned to decrease administrative workload and mitigate burnout-related risks. These strategies are essential for ensuring data accuracy as the registry transitions toward a sustainable, mandatory national platform.

Looking ahead, the AAMATES registry is positioned to evolve beyond its cross-sectional structure through the incorporation of longitudinal clinical outcomes. Planned enhancements include the systematic capture of dialysis technique failure, transitions between modalities, kidney transplantation, and all-cause and cause-specific mortality. Future iterations will also integrate hospitalizations, cardiovascular events, vascular access outcomes, infectious complications, and patient-reported outcomes. These developments will transform AAMATES into a dynamic, outcomes-based registry, substantially strengthening its capacity to inform clinical care, health system performance, and policy planning in Mexico.

In conclusion, this first evaluation of the AAMATES registry of individuals with KDIGO stage 5 CKD offers insight into the current management of advanced CKD in CCINSHAE hospitals. Although it represents a significant advance, further development and external validation with other national health care institutions remain necessary.
